# Supplementary material for: SurvivalPath:A R package for conducting personalized survival path mapping based on time-series survival data
Source: PLoS Comput Biol. 2023 Jan 6;19(1):e1010830. doi: 10.1371/journal.pcbi.1010830 (PMC9851545; doi:10.1371/journal.pcbi.1010830)
Supplement: Attachment — Submitted filename: Response to reviewers (Rebuttal letter).docx [file pcbi.1010830.s001.docx]

Dear editor and reviewers,

Thank you very much for your comments on our manuscript of “SurvivalPath:A R package for conducting personalized survival path mapping based on time-series survival data” **(No:PCOMPBIOL-D-22-01103)***.* We have carefully responded to the comments by reviewers. Besides, we corrected several typing errors in the previous manuscript. We believe that our paper is significantly improved after the revision.

Listed below are our point-by-point responses to the critiques of the reviewers. We hope that the revised manuscript will meet your approval for publication.

**Replies to Reviewer #1**:

**The auther develop a SurvivalPath R package to facilitate building personalized survival path models. The package contains functions to convert time-series data by into time-slices data by fixed interval based on time information of input medical records. And I have noticed that this R package is based the work which has published. However, I still have some questions:**

**1: How does the SurvivalPath R package deal with the missing data?**

**Answer:** Many thanks for this advice. In the function generatorDTSD() of our package, there is an argument “isfill”, select TURE, then the missing value of variables in specific time slice will be filled by the mean value of that variable in that time slice. The package now didn’t contain other filling methods, if the user wants to use other filling methods to deal with missing values, he may have to fill his dataset through other tools himself.

As reminded by the reviewer, we will add an additional functions on data washing and missing data filling in the next version of the SurvivalPath R package.

2. **In Figure 2, I want to know that whether SurvivalPath R package is suitable for time-series data with a three-class outcome(eg: alive, progress, death) and one continuous variable(eg: ALBI score)**

**Answer:** Many thanks for this important question. The current methodology for ensemble modeling is built based on Cox regression model. The primary outcome of modeling need to contain event and time, and event has to be coded as binary variable (e.g. 0,1). It’s a pity that this model does not apply for predicting a continuous variable, which is also not the objective of this methodology. It’s also an interesting field of predicting ALBI score dynamically using time-series data after consequential treatments, including interventional treatments, surgery and so on; and this field worth our future efforts.

**3: In Figure 3, graph of survival path is too complex for doctor to use. If it is possible, the number of survival path should be less than 5.**

**Answer:** Many thanks for this kind advice. The Figure 3 is a demo graph using built-in dataset. The user can build personalized survival path through the survivalpath() function by setting parameters including *minsample*, *P value*. Increasing *minsample* for bifurcation and selecting smaller *P value* will produce less complex survival path graph. Meanwhile, increase the interval of each time-slice during the “time division” period will generate dataset with less time slices, which will facilitate building more concise and simple survival path graphs.

**Replies to Reviewer #2:**

**The authors submit an interesting manuscript (PCOMPBIOL-D-22-01103) about conducting personalized survival path mapping based on time-series survival data for possible publication in PLOS Computational Biology.**

**1.The authors introduced the SurvivalPath R package for dynamic prognostication of cancer patients using time-series survival data. I have installed the SurvivalPath R package, and verified that it works to facilitate building personalized survival path models. Congratulations to them!**

**Answer:** Many thanks for this acknowledgement!

**2.I believe that readers could benefit from this package which contains functions to convert time-series data by into time-slices data by fixed interval based on time information of input medical records. More importantly, under a user-defined parameters on covariates, significance level, minimum bifurcation sample size and number of time slices for analysis, survival paths can be computed using the main function, which can be visualized as a tree diagram, with important parameters annotated. Besides, the package can also be used for further exploration analysis, which I think is an important application of this methodology.**

**Answer:** Many thanks for this comment.

**3.One concern is how this package deal with the situations that specific variable with multiple important cutoffs. I notice that the optimal cutoff of continuous variable could be automatically computed, while for different situations and in different time slices, the optimal cutoff could be different.**

**Answer:** This is an very important issue when designing this package. For continuous variables, optimal cutoff will be automatically computed; if the continuous variable have multiple cutoff of interest and all of them need to be included into building the survival path model, the researcher need to add the binary variables with each specific cutoff separately into the time-series dataset before computing the survival path model.

**4.Generally, the manuscript is well organized and easy to follow. I think it can be accepted after language polishing.**

**Answer:** Many thanks for this acknowledgement!
